# Structure of Reovirus σ1 in Complex with Its Receptor Junctional Adhesion Molecule-A

**DOI:** 10.1371/journal.ppat.1000235

**Published:** 2008-12-12

**Authors:** Eva Kirchner, Kristen M. Guglielmi, Holger M. Strauss, Terence S. Dermody, Thilo Stehle

**Affiliations:** 1 Interfaculty Institute for Biochemistry, University of Tuebingen, Tuebingen, Germany; 2 Department of Microbiology and Immunology, Vanderbilt University School of Medicine, Nashville, Tennessee, United States of America; 3 Elizabeth B. Lamb Center for Pediatric Research, Vanderbilt University School of Medicine, Nashville, Tennessee, United States of America; 4 Nanolytics Gesellschaft für Kolloidanalytik mbH, Potsdam, Germany; 5 Department of Pediatrics, Vanderbilt University School of Medicine, Nashville, Tennessee, United States of America; Institut Pasteur, France

## Abstract

Viral attachment to specific host receptors is the first step in viral infection and serves an essential function in the selection of target cells. Mammalian reoviruses are highly useful experimental models for studies of viral pathogenesis and show promise as vectors for oncolytics and vaccines. Reoviruses engage cells by binding to carbohydrates and the immunoglobulin superfamily member, junctional adhesion molecule-A (JAM-A). JAM-A exists at the cell surface as a homodimer formed by extensive contacts between its N-terminal immunoglobulin-like domains. We report the crystal structure of reovirus attachment protein σ1 in complex with a soluble form of JAM-A. The σ1 protein disrupts the JAM-A dimer, engaging a single JAM-A molecule via virtually the same interface that is used for JAM-A homodimerization. Thus, reovirus takes advantage of the adhesive nature of an immunoglobulin-superfamily receptor by usurping the ligand-binding site of this molecule to attach to the cell surface. The dissociation constant (K_D_) of the interaction between σ1 and JAM-A is 1,000-fold lower than that of the homophilic interaction between JAM-A molecules, indicating that JAM-A strongly prefers σ1 as a ligand. Analysis of reovirus mutants engineered by plasmid-based reverse genetics revealed residues in σ1 required for binding to JAM-A and infectivity of cultured cells. These studies define biophysical mechanisms of reovirus cell attachment and provide a platform for manipulating reovirus tropism to enhance vector targeting.

## Author Summary

Mammalian orthoreoviruses (reoviruses) are useful models for studies of virus–receptor interactions and viral pathogenesis. They are closely related in structure to adenoviruses and share similar mechanisms of cell attachment and entry. The receptor for reovirus, junctional adhesion molecule-A (JAM-A), is a component of cellular junctions and also used as a receptor by feline calicivirus. To better understand how viruses engage cellular receptors, we determined the structure of reovirus attachment protein σ1 bound to JAM-A. The structure provides an understanding of the biological function of the interaction and yields information that may enable targeting of reovirus to alternate receptors. Since the repertoire of receptors bound by a virus contributes importantly to determining which types of cells are infected, such targeting plays an essential role in gene delivery for vaccine or therapeutic applications. New cancer therapy approaches include the use of viruses, including reovirus, to lyse tumor cells. New knowledge about reovirus attachment to cellular receptors at an atomic level will help to harness the therapeutic potential of this virus.

## Introduction

Viruses have evolved a variety of strategies to engage cellular receptors, often taking advantage of the adhesive properties of these molecules. Immunoglobulin superfamily (IgSF) members mediate cellular adhesion functions including antigen recognition, stabilization of intercellular junctions, adhesion to extracellular matrices, and leukocyte extravasation [1]. These cell-surface proteins are also used as receptors by many viruses [2],[3]. Junctional adhesion molecule-A (JAM-A) is an IgSF member that mediates cell-cell contacts and serves as a receptor for mammalian orthoreovirus (reovirus) [4] and feline calicivirus [5]. Reovirus serves as a tractable experimental model for studies of virus-receptor interactions and viral pathogenesis. Virtually all mammals including humans serve as hosts for reovirus infection, but disease is restricted to the very young [6]. The recent development of plasmid-based reverse genetics for reovirus offers the opportunity to manipulate these viruses for oncolytic and vaccine applications [7].

Reoviruses form icosahedral particles approximately 850 Å in diameter [6]. At the virion fivefold symmetry axes, the trimeric attachment protein, σ1, extends from pentameric turrets formed by the λ2 protein [8],[9]. A similar arrangement of a trimeric attachment protein inserted into a pentameric base is also observed for the adenovirus attachment protein, fiber [10]. The σ1 molecule is about 480 Å in length and composed of a filamentous N-terminal tail and a globular C-terminal head [8],[9]. Discrete regions of the molecule mediate binding to cell-surface receptors. Sequences in the tail bind to carbohydrate [11], which is α-linked sialic acid for serotype 3 reoviruses [12]. The σ1 head binds to JAM-A [4],[13].

Structural analysis of the C-terminal region of strain type 3 Dearing (T3D) σ1, which includes the region that binds to JAM-A [4], has revealed details of its trimeric structure [13],[14]. Residues forming the head consist of two Greek-key motifs that fold into a compact β-barrel. The topology of this structure is identical to the β-sandwich that forms the receptor-binding knob of adenovirus fiber, pointing to a distant evolutionary relationship between the two proteins [14]. Loops connecting individual strands of the σ1 β-barrel are short with the exception of the D–E loop (connecting β-strands D and E), which contains a 3_10_ helix. N-terminal residues in the crystallized fragment form a portion of the tail, which consists of three triple β-spiral repeats. To date, the triple β-spiral motif has been observed only in adenovirus fiber [15], bacteriophage PRD1 spike [16], and avian reovirus attachment protein σC [17].

JAM-A is an important component of tight junctions between endothelial and epithelial cells [18],[19]. It is also expressed on the surface of platelets and leukocytes [20]. JAM-A influences the migration of leukocytes across endothelial and epithelial barriers in response to inflammatory cues [21],[22]. The extracellular portion of JAM-A forms a homodimer in which the monomers are partially intertwined via interactions of the membrane-distal D1 domains [23],[24]. Interestingly, the only other example of structurally similar homodimeric interactions by an IgSF member is the coxsackievirus and adenovirus receptor, CAR [25].

Domain-swapping experiments indicate that the D1 domain of JAM-A is necessary for functional interactions with reovirus [26]. Thus, our efforts to identify σ1-binding regions in JAM-A have focused on D1. Biochemical studies have identified the dimer interface as the region of JAM-A bound by reovirus σ1, and individual residues in JAM-A that are required for efficient σ1 binding are located within this interface [24],[26],[27]. In addition, complexes formed between purified σ1 head domain and purified dimeric wild-type (wt) or monomeric point-mutant forms of JAM-A are indistinguishable by size-exclusion chromatography [27], suggesting that a monomeric form of JAM-A serves as the relevant binding partner for σ1.

To define the structural basis of σ1-JAM-A interactions, we crystallized a complex of the head domain of T3D σ1 and the D1 domain of human JAM-A (hJAM-A) and determined its structure at 3.4 Å resolution. Since σ1 binds to a monomeric form of JAM-A, we determined the dissociation constant (K_D_) of the homophilic JAM-A interaction by analytical ultracentrifugation to define the stability of the JAM-A dimer and the mechanism of σ1-JAM-A complex formation. Finally, we used plasmid-based reverse genetics to engineer reoviruses expressing mutant forms of σ1 to determine the contributions to binding and infectivity of specific residues that contact JAM-A. These studies reveal the biochemical basis of σ1-JAM-A interactions, provide clues about how σ1 successfully competes for the JAM-A dimer interface, and establish a platform for fine-tuning receptor recognition to enhance the targeting of reovirus vectors.

## Results

### Complex Formation and Crystallization

A T3D σ1 fragment comprising the head domain and one β-spiral of the tail (σ1H; residues 293–455) and the D1 domain of hJAM-A (D1; residues 28–129) were purified using glutathione S-transferase (GST)-affinity purification [13],[27]. The domain boundaries were chosen to eliminate regions of known flexibility [14],[24] and retain binding capacity [13],[24],[27]. Purified σ1H was mixed with an excess of D1 to ensure saturation of binding. Following incubation, σ1H-D1 complexes were separated from excess D1 by size-exclusion chromatography and crystallized.

The structure of the σ1H-D1 complex was determined by molecular replacement and refined to 3.4 Å resolution (Table 1). The crystallographic asymmetric unit consists of two σ1H trimers, each bound to three D1 monomers. The presence of six independent copies enabled us to carry out six-fold non-crystallographic averaging of the components and refinement using non-crystallographic symmetry restraints. These techniques helped to establish a reliable model in which the main chain and most of the side chains, including those at the contact interface, are defined by satisfactory electron density. Real-space correlation plots show that the structure is in good agreement with the electron density (Figure S1). The dataset was assembled from three individual crystals, which may explain the relatively high merging *R*-factor of 16.3% (*R*_merge_, Table 1). In contrast, the refinement *R*-factor is relatively low at 21.0% (*R*_work_, Table 1). Because of sixfold non-crystallographic symmetry in the crystals, our free set of reflections, used as a control for the *R*-factor during refinement, is most likely not totally “free.”

**Table 1:** Data collection and refinement statistics.

**Data collection** a	
Space group	P2_1_2_1_2
Unit cell dimensions (Å)	*a* = 105.9, *b* = 124.3, *c* = 130.6
Resolution (Å)	30.00–3.40 (3.52–3.40)^b^
*R*_merge_ (%)^c^	16.3 (21.2)
*I/σI*	6.9 (2.7)
Completeness (%)	90.2 (69.7)
Redundancy	3.6 (1.9)
*B*-factor from Wilson plot (Å^2^)	64.5
**Refinement**	
Resolution (Å)	30.00–3.40 (3.52–3.40)
No. reflections	21,954
*R*_work_ / *R*_free_ (%)^d^	21.0 (28.4) / 25.2 (32.8)
No. atoms	12,227
*B*-factor (Å^2^), overall	62.1
*B*-factor (Å^2^), σ1H	56.1
*B*-factor (Å^2^), D1	71.8
R.m.s.d. Bond lengths (Å)	0.011
R.m.s.d. Bond angles (°)	1.544
*Ramachandran plot:* e	
Residues in favoured region (%)	91.2
Residues in allowed region (%)	8.8
Residues in outlier region (%)	0.1

a Three crystals were used to assemble the dataset.

b Values in parentheses are for highest resolution shell.

c *R*_merge_ = ∑*_hkl_* |*I−<I>*|/∑*_hkl_ I*, where *I* is the intensity of a reflection *hkl*, and *<I>* the average over symmetry-related observations of *hkl*.

d *R*_cryst_ = ∑*_hkl_* |*F*_obs_−*F*_calc_|/∑*_hkl_ F*_obs_, where *F*_obs_ and *F*_calc_ are observed and calculated structure factors, respectively. Free set [65] contains 10% of the data. Free reflections were selected randomly.

e Calculated with Rampage [55].

### Overall Structure of the Complex

**Figure 1:**
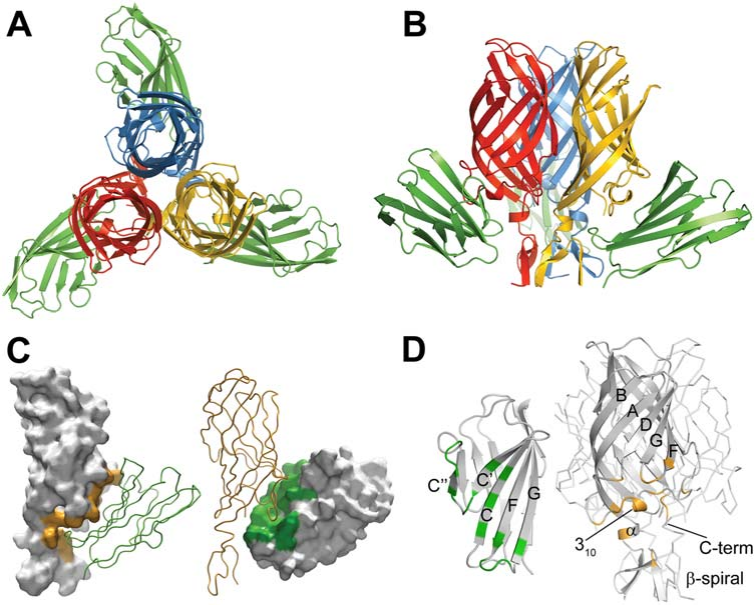
Structure of the σ1H-D1 complex. (A,B) Ribbon drawings of the complex between trimeric σ1H and monomeric D1, viewed along the three-fold symmetry axis (A) and from the side (B). σ1H monomers are shown in blue, yellow, and red; D1 is shown in green. (C) Surface representation of the contact area of reovirus σ1H (left, orange) and D1 (right, green). Interacting partners are shown as ribbon traces. (D) Ribbon drawings of D1 (left) and σ1H (right). Secondary structure elements are labeled. Contact residues (distance cut-off 4 Å) in the σ1H-D1 interface are colored green (D1) or orange (σ1H).

The crystallized complex consists of a σ1H trimer ligated by three D1 monomers. When viewed along the three-fold non-crystallographic symmetry axis, its overall structure resembles a three-bladed propeller, with σ1H forming the hub and D1 forming the blades (Figure 1A). Each D1 monomer interacts with one σ1H monomer, making extensive contacts that shield a combined area (the sum of contact areas on both proteins) of 1622 Å^2^ from solvent. Crystal packing results in additional contacts between the molecules. However, the interactions we describe are common to all σ1H-D1 pairs and likely represent the physiologic complex interface. D1 residues involved in contact formation are located at the most membrane-distal (top) part of the domain and on the face that mediates homodimer formation. These regions in D1 pack tightly into a recessed region of σ1H just below the β-barrel (Figure 1B and 1C). Residues at the D1 dimer interface form extensive contacts with the D–E loop and 3_10_ helix of σ1H at the upper boundary of the recessed region, whereas the top of D1 contacts residues in the β-spiral of the σ1H tail at its lower boundary. In comparison to structures of isolated σ1 [13],[14] and hJAM-A [24], the architecture of both σ1H and D1 in the complex are largely preserved. Differences are observed primarily in side-chain orientations at the interfaces between σ1H and D1.

Four of the six σ1H-D1 pairs present in the asymmetric unit have similar structures and feature the same interactions. The analysis of the complex presented here is based on these pairs. The remaining two σ1H-D1 pairs exhibit larger intermolecular distances of up to 1.2 Å, resulting in fewer contacts and higher crystallographic temperature factors. The total buried surface area for these two interacting pairs is about 60 Å^2^ less. Crystal packing is very tight for a protein complex of this size, with only 50% solvent content [28]. The largest gaps in the packing occur directly beneath the D1 chains that exhibit larger intermolecular distances to σ1H. Flash-cooling of crystals prior to data collection may have partially dislodged D1 from its binding site at these locations [29].

### Interaction of Reovirus σ1H with JAM-A D1

**Figure 2:**
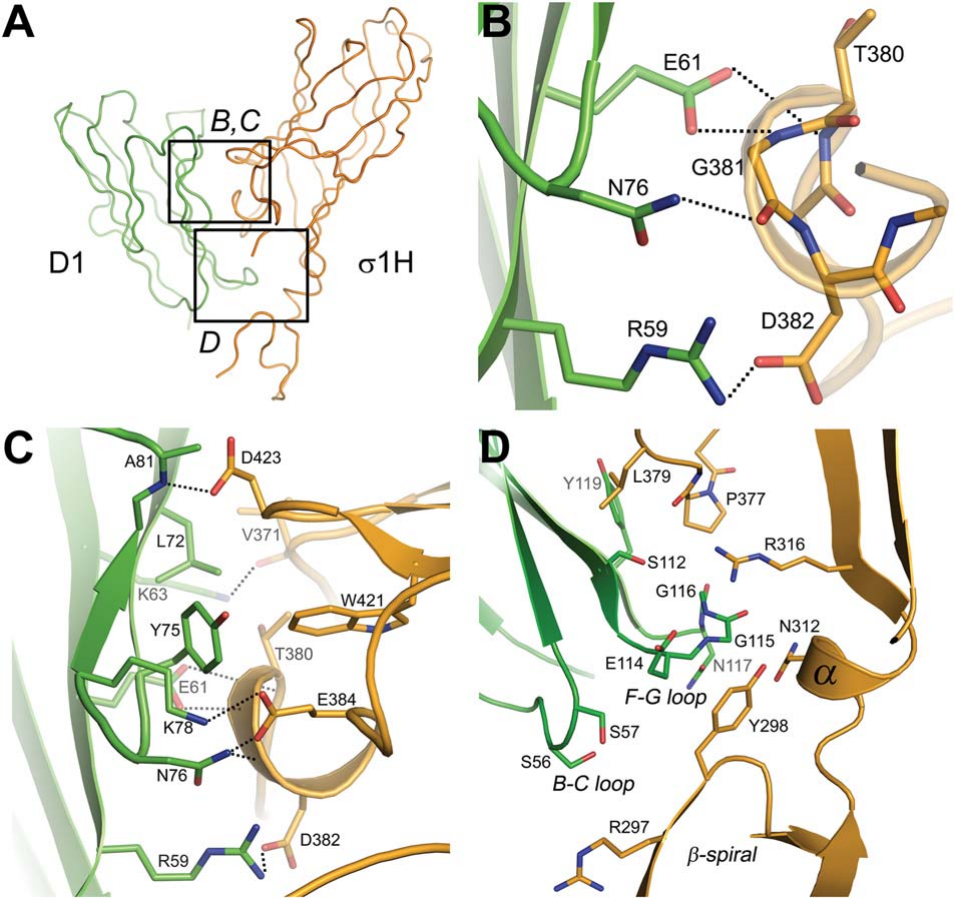
Contacts at the σ1H-D1 interface. (A) Overview displaying the location of residues in the σ1H-D1 complex shown in (B) and (C) (upper jaw) and (D) (lower jaw). D1 and σ1H are colored green and orange, respectively. (B–D) Carbon atoms are shown in green (D1) or orange (σ1H), oxygen atoms in red, and nitrogen atoms in blue. Dotted lines represent hydrogen bonds and salt bridges. For clarity, only interacting residues are shown. Amino acids are labeled in single-letter code. (B) Interactions between D1 and residues in the 3_10_ helix in the D–E loop of σ1H. The 3_10_ helix is depicted transparently so that the main chain interactions are visible. (C) Additional interactions between D1 and σ1H around the region depicted in B. (D) Interactions between the F–G and B–C loops of D1 with σ1H.

**Figure 3:**
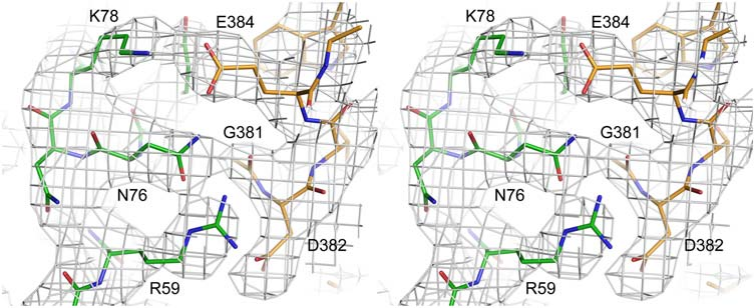
Composite annealed omit map of a σ1H-D1 contact area. 2F_obs_-F_calc_ composite annealed omit map, calculated with CNS [56], contoured at 1σ and shown in stereo. The map depicts key residues and their interactions at the complex interface. Carbon atoms of D1 and σ1H are shown in green and orange, respectively. Oxygen atoms are shown in red and nitrogen atoms in blue. Amino acids are labeled in single-letter code.

Reovirus σ1H engages JAM-A D1 using two main contact areas: a larger region centered at the D–E loop and its 3_10_ helix, just below the β-barrel, and a smaller region formed by the top of the β-spiral and the α-helix (Figure 1D). These two regions resemble “jaws” that grip the D1 domain at its interdomain interface and top (Figure 2A). Although exact placement of individual atoms is not possible at 3.4 Å resolution, there is unambiguous electron density in an omit map for all side chains in the interface (Figure 3), allowing for assignment of contacts.

The upper, larger σ1H jaw contacts the D1 interdomain interface. Contacts are largely polar, featuring numerous hydrogen bonds and two salt bridges. These interactions are centered at the σ1H 3_10_ helix, in which residues Thr380, Gly381, and Asp382 interact with D1 residues Glu61, Asn76, and Arg59, respectively (Figure 2B). These contacts are augmented by interactions between σ1H D–E loop residues Val371 and Glu384 and D1 residues Asn76, Lys78, and Lys63, and by contacts between Asp423 in the F–G loop of σ1H and the main-chain nitrogen atom of Ala81 (Figure 2C). In addition to these polar interactions, D1 residues Leu72 and Tyr75 engage in hydrophobic contacts with D–E loop residues and the terminal part of β-strand F in σ1H (Figure 2C). Previous point mutagenesis studies indicate that D1 residues Arg59, Glu61, Lys63, Leu72, Tyr75, and Asn76 contribute to σ1 binding [27]. Interestingly, most of the D1 residues engaged in interactions with σ1H form contacts of a similar nature in the JAM-A homodimer. For example, D1 residue Arg59 forms a salt bridge with Asp382 in the complex and a salt bridge with D1 residue Glu61 in the JAM-A dimer. Similarly, Leu72 and Tyr75, which mediate hydrophobic contacts in the complex, also do so in the JAM-A dimer.

Contacts mediated by the smaller, lower jaw of σ1H lack hydrogen bonds and salt bridges. Instead, extensive hydrophobic interactions with substantial surface complementarity are found, indicating that this area also plays an important role in defining specificity and providing high affinity. In σ1H, interactions involve β-spiral residue Tyr298, a mostly hydrophobic surface of the α-helix connecting the β-spiral with the β-barrel, the non-polar portion of the Arg316 side chain, and Pro377 in the D–E loop (Figure 2D). These residues surround the D1 F–G loop, which contains several partially hydrophobic residues. The nearby B–C loop of D1 also faces towards the σ1H β-spiral, with its closest contact between the hydroxyl group of D1 residue Ser57 and the tip of the β-spiral in σ1H. Ser57 also contributes to σ1 binding [26].

**Figure 4:**
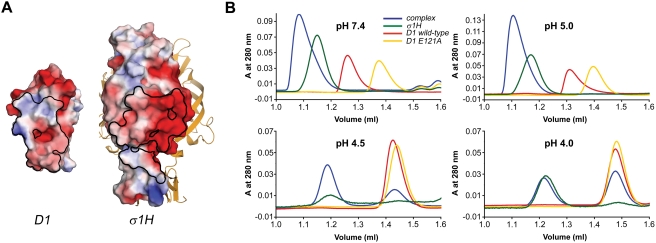
Electrostatic potentials and complex stability at low pH. (A) Electrostatic potential of the surfaces of D1 (left) and a single σ1H subunit (right) calculated with APBS tools [64]. The scale ranges from −3 (red) to +3 (blue) in units of k_B_T/e_c_. Boundaries of the contact areas of the complex are outlined in black. The other two σ1H monomers are shown as yellow ribbons. (B) Size-exclusion chromatographs of the σ1H-D1 complex using conditions of varied pH. The σ1H, wt D1, and monomeric D1 E121A proteins were used as controls to determine whether a shift in elution volume was attributable to disassociation of the complex into its components or pH-dependent alteration of protein elution behavior. Glu121 in the D1 dimer interface does not participate in complex formation with σ1H. Thus, its alteration affects JAM-A dimerization but not σ1H ligation [27]. The σ1H-D1 complex was stable at pH values 7.4 and 5.0, eluting from the column earlier than σ1H, wt D1, and D1 E121A. However, at pH values 4.5 or 4.0, the complex dissociated into its components, which eluted at the same volumes as the controls, σ1H and D1. Similarly, wt D1 dissociated under conditions of low pH, eluting at the same volume as the monomeric mutant D1 E121A. This result is in agreement with data obtained in previous studies of the murine JAM-A dimer [30]. At pH 4.5 and 4.0, the A_280_ of σ1H was multiplied by 10 to compensate for the lower concentration due to precipitation.

The majority of interactions between σ1H and D1 involve hydrophilic residues, with a surprisingly large number of charged residues participating in contact formation. Three charged σ1H residues directly mediate polar interactions with D1, and two others do so indirectly. In D1, four direct contacts are formed with charged residues. As a result, the interacting surfaces of both σ1H and D1 display strong electrostatic potentials (Figure 4A). When comparing the two, the interacting surface of σ1H has a dominant electronegative potential in the upper jaw, whereas the lower jaw is electropositive. The interacting surface of D1 is complementary to σ1H, featuring an electropositive potential at the dimer interface and a more electronegative potential at the most membrane-distal part of the domain. The importance of charged residues in the interaction between σ1 and JAM-A is highlighted by the observation that the complex dissociates at pH values lower than 5 (Figure 4B).

### Stability of the JAM-A Homodimer

The σ1H-D1 complex is readily produced in solution by mixing the two components. Although JAM-A dissociates under high salt or low pH conditions [30], we were not able to detect monomeric species of JAM-A in the neutral pH, low salt conditions used for complex formation (data not shown). Thus, we conclude that complex formation requires disruption of JAM-A homodimers by σ1. This process could be facilitated by a significantly higher affinity between σ1 and JAM-A D1 compared to that of the homophilic JAM-A interaction. The dissociation constant (K_D_) for the σ1-JAM-A complex is in the low nanomolar range [4],[27]. To determine a K_D_ value for the JAM-A D1 homodimer, we performed analytical ultracentrifugation experiments at near-physiological conditions (Tris pH 7.5, 100 mM NaCl). Five JAM-A D1 samples at concentrations ranging from 0.06 to 1.31 mg/mL were used for the sedimentation velocity experiments. Sedimentation velocities showed little concentration dependence of the sedimentation coefficient (Figure S2A). The main component sediments at ∼2.35 S. This value corresponds to molar masses between 19 kg/mol and 22 kg/mol, close to that expected for dimeric JAM-A D1. We also detected significant but variable amounts of a second component, sedimenting at 3.8 S. This species is most likely tetrameric JAM-A D1. While tetramers of JAM-A in solution have been observed [30], our analytical ultracentrifugation experiments did not reveal a tendency of JAM-A to form tetramers in a concentration-dependent manner, suggesting that this species is not physiologic.

Sedimentation equilibrium experiments were conducted at four different concentrations (0.16 to 1.6 mg/mL) at three different speeds. The best fit (r.m.s.d. of 1.99×10^−2^ with 5109 degrees of freedom) for all available data sets was for a monomer-dimer model with variable amounts of tetramer (Figure S2B). The molar mass converged to a value of 10.94 kg/mol (10.90 to 11.16 kg/mol), which is very close to the expected molar mass for monomeric JAM-A D1 (11.5 kg/mol). The K_D_ for this fit is 1.1×10^−5^ M (0.8 to 1.4×10^−5^ M). If the molar mass is constrained to the expected value, a poorer fit (r.m.s.d. error of 2.19×10^−2^, 5110 degrees of freedom) is obtained. The slight mismatch between the best-fit and the expected molar mass indicates an imprecision in the calculation of the partial specific volume or density of the buffer.

### Contribution of Individual σ1 Residues to JAM-A Engagement and Infectivity

To identify contributions of individual residues in σ1 to JAM-A engagement, we employed plasmid-based reverse genetics [7] to engineer mutations into the σ1 protein of reovirus strain T3D. Mutant viruses were isolated following co-transfection of murine L929 (L) cells with nine RNA-encoding plasmids corresponding to wt T3D genes and a tenth plasmid corresponding to the σ1-encoding *S1* gene incorporating site-specific mutations. Thus, each resultant virus is isogenic, with the exception of the *S1* gene and its protein product, σ1. Guided by the structure of the σ1H-D1 complex, we engineered individual substitutions of Thr380, Gly381, and Glu384 in the D–E loop and Asp423 in the F–G loop of the JAM-A-binding region of σ1. In addition, we also mutated Asn369, which is located at the N-terminus of the D–E loop, but does not contact JAM-A. With the exception of Asp423, these residues are conserved in sequence alignments among prototype strains from all three reovirus serotypes [14]. All mutant viruses were recovered and produced sufficient titer to allow binding and infectivity studies.

**Figure 5:**
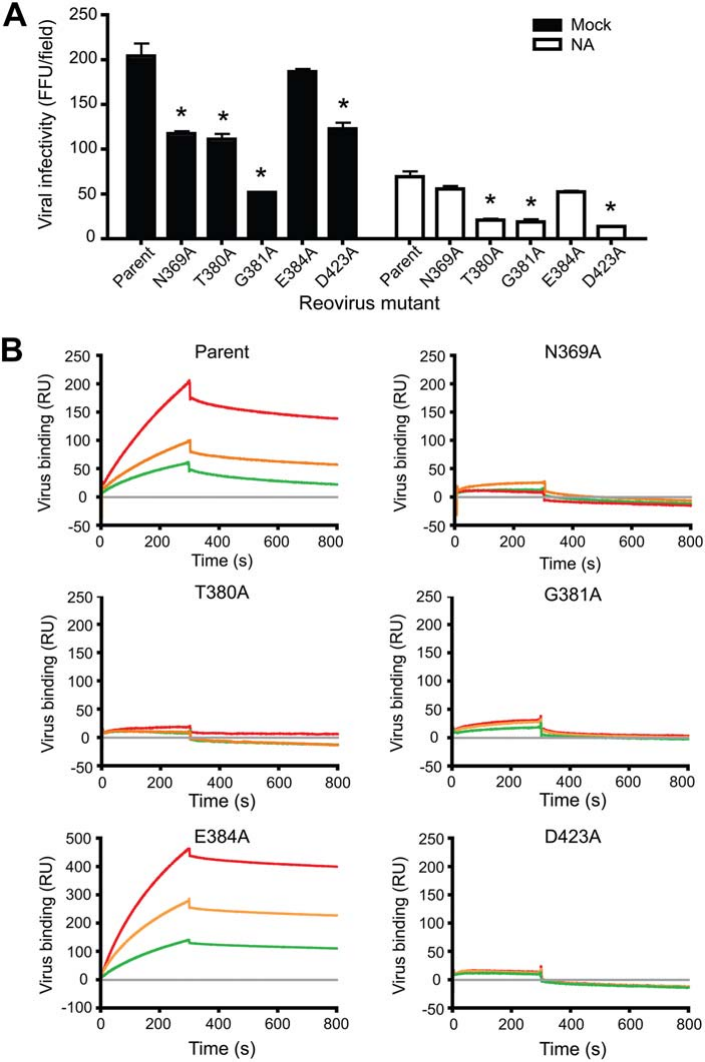
Identification of σ1 residues required for infection of cultured cells and JAM-A binding. (A) Infection of HeLa cells by parent or mutant virus. Cells were treated with either PBS alone (mock) or 40 mU/ml of neuraminidase diluted in PBS (NA). Infected cells were identified by indirect immunofluorescence and quantified in three fields of view. The results are expressed as the mean fluorescent focus units (FFU) per field for triplicate experiments. Error bars indicate standard deviations. The asterisk indicates *P*<0.05 in comparison to the control. (B) SPR analysis of reovirus binding to JAM-A. Traces show binding and dissociation from GST-JAM-A for purified reoviruses at a concentration of 6×10^12^ (green), 8×10^12^ (orange), and 10^13^ (red) particles/ml. Reovirus binding to the capturing molecule (grey) is set as the baseline. Binding is expressed in resonance units (RU).

To determine effects of substitutions in the JAM-A-binding region of σ1 on viral infectivity, we adsorbed HeLa cells with the parent or mutant viruses at a multiplicity of infection (MOI) of 50 plaque-forming units (PFU) per cell and quantified infected cells in confluent fields of view following 20 h of incubation. With the exception of E384A, each of the point-mutant viruses exhibited significantly diminished infectivity in comparison to the parent strain, with the G381A mutant infecting the fewest cells (Figure 5A). T3 reoviruses bind to sialic acid, an event mediated by sequences in the σ1 tail [11],[31], which enhances attachment and infectivity in HeLa cells [11],[31]. Therefore, the parent and σ1 point-mutant viruses should retain the capacity to bind sialic acid. To determine effects on viral infectivity of mutated residues in the JAM-A-binding surface of σ1 in the absence of sialic acid binding, we pre-treated HeLa cells with *A. ureafaciens* neuraminidase to remove sialic acid prior to viral adsorption (Figure 5A). As expected, neuraminidase-treatment resulted in decreased infectivity for all viruses, with ∼60% fewer infected cells for the parent virus. In comparison to the parent strain, the T380A, G381A, and D423A viruses exhibited a significant decrease in viral infectivity in the absence of sialic acid. The relative decrease in infectivity of N369A compared to the parent virus following neuraminidase treatment was less than that observed in untreated cells. The explanation for this result is not clear, but it may be due to some type of cooperative interaction between the σ1 receptor-binding domains unmasked by the N369A mutant. We conclude that targeted mutations in the JAM-A-binding surface of σ1 influence viral infectivity, presumably due to altered viral avidity for JAM-A.

To determine the JAM-A-binding capacity of the mutant viruses, we captured purified JAM-A, as an N-terminal fusion with GST, on a biosensor surface and employed surface plasmon resonance (SPR) to assess viral binding [27]. Upon injection of the parent virus at 6×10^12^, 8×10^12^, and 1×10^13^ particles/mL, we observed specific, concentration-dependent association with JAM-A over time (Figure 5B). In accord with the infectivity results, all mutant viruses except E384A exhibited diminished binding in comparison to the parent strain, suggesting that these residues contribute significantly to interactions with JAM-A. Interestingly, the E384A mutant exhibited higher overall binding responses than the parent virus, suggesting this virus has enhanced avidity for JAM-A. However, this enhanced avidity does not appear to translate into enhanced infectivity in HeLa cells (Figure 5A).

## Discussion

**Figure 6:**
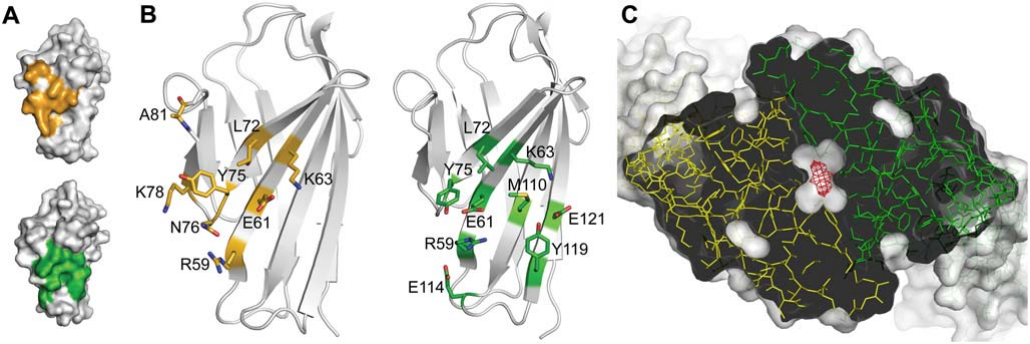
Comparison of the JAM-A D1 dimer and the σ1H-D1 complex. (A) Surface representations of D1, with key contact residues (residues forming hydrogen bonds, salt bridges, or close hydrophobic contacts) highlighted in orange (σ1H-D1 complex, top) or green (D1 dimer, bottom). Additional contacts are not shown. (B) Ribbon drawings of D1 showing stick representations of the same contacts depicted in (A). Carbon atoms are shown in orange (σ1H-D1 complex, left) or green (JAM-A dimer, right), oxygen atoms in red, nitrogen atoms in blue, and sulfur atoms in yellow. Amino acids are labeled in single-letter code. (C) Cavity at the JAM-A homodimer interface. The JAM-A homodimer [24] is viewed along the two-fold axis and depicted in stick representation (green and yellow). The protein surface is shown in a semitransparent white rendering. The JAM-A D1 domain is opened at the center to reveal the cavity, calculated using VOIDOO [33], which is shown as a red mesh.

The interaction between reovirus σ1 and JAM-A is the first step in an infectious cycle that culminates in the death of the target cell. While some reovirus strains use additional co-receptors, all strains engage JAM-A [32]. JAM-A exists as a dimer in solution [30] and most likely at the cell surface, but monomers are bound by σ1 in our crystal structure. The binding studies we report here show that formation of the σ1-JAM-A complex is clearly preferred to the formation of JAM-A homodimers. The interaction between two JAM-A molecules has a K_D_ of 1.1×10^−5^ M, whereas the K_D_ for the σ1-JAM-A interaction is about 1,000-fold lower [27]. These differences in affinity are remarkable given that the surfaces buried in the two complexes are strikingly similar in shape, almost identical in size, and share many of the same residues (Figure 6A and 6B). Why might JAM-A have a higher affinity for σ1 than for JAM-A? The structure of the JAM-A dimer [24] reveals a cavity in the dimer interface of about 6.9 Å^3^ in size (Figure 6C) (calculated using VOIDOO [33]). In contrast, no cavities are found in the six copies of the σ1-JAM-A complex interfaces, which feature nearly perfect surface complementarity. Cavities in protein-protein interfaces usually contain water molecules that can significantly destabilize hydrogen bonds and salt bridges by lowering the dielectric constant of the medium. Indeed, two water molecules are visible in the cavity of the JAM-A dimer interface, and two more are adjacent to this surface [24]. The presence of water at the center of the JAM-A dimer interface could thus weaken the homophilic interaction. Concordantly, the JAM-A dimer interface is dynamic, which is thought to facilitate transitions between monomeric and dimeric forms [24]. The transitional nature of the homophilic JAM-A interaction may play a role in the regulation of tight junction permeability. A similar cavity is found in the crystal structure of murine JAM-A [23], which also can bind σ1 [4].

Our results indicate that several residues in the σ1 D–E loop are especially important for efficient JAM-A engagement. Mutation of Asn369, Thr380, Gly381, or Asp423 to alanine leads to drastically impaired JAM-A binding on a biosensor surface and reduced infectivity of HeLa cells (Figure 5). These results can now be rationalized by the structure of the complex. Mutation of Gly381 would adversely affect interactions with JAM-A, as any side chain at this position would lead to steric clashes with D1 residue Tyr75. The Thr380 side chain likely shields hydrophobic interactions from solvent (Figure 2C). Moreover, since Thr380 makes extensive contacts with other σ1 residues, truncation of its side chain would likely affect the structural integrity of the 3_10_ helix and thus diminish JAM-A binding. Changes in local structure also might explain the reduced binding observed for the N369A mutant. Although Asn369 does not directly contact D1, its location at the N-terminus of the D–E loop may help to stabilize the 3_10_ helix. Asp423 interacts with the main chain amide group of Ala81 in JAM-A and, like Thr380, shields hydrophobic interactions from solvent. Interestingly, the E384A mutant exhibits slightly enhanced binding to JAM-A. The Glu384 side chain interacts with nearby σ1 residues His388 and Trp421 and may stabilize this region, which probably includes several water molecules bound to surrounding side chains. These interactions are likely altered to allow σ1 to bind JAM-A. We think it possible that truncation of the Glu384 side chain would facilitate this process.

**Figure 7:**
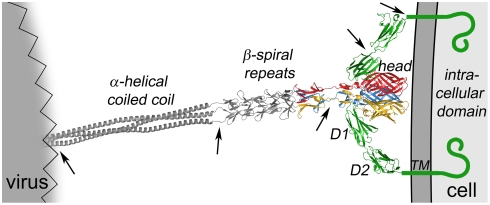
Full-length model of the σ1-JAM-A complex. A model of full-length σ1 extending from a schematic representation of a virion is shown as a ribbon drawing, with the known structure of the C-terminus [14] in tricolor and the predicted structure of the N-terminus in grey. A model of full-length JAM-A is shown in green as a ribbon drawing of the known structure of the extracellular domain [24] and a schematic representation of the transmembrane (TM) and intracellular domains. Arrows indicate regions of flexibility. For clarity, only two JAM-A monomers are shown bound to σ1.

To visualize how σ1 interacts with JAM-A at the cell surface, we combined the structures of the σ1H-D1 complex, the JAM-A extracellular domain [24], and the C-terminus of σ1 [14] with a model of the N-terminus of σ1 [14],[34], as previously done to generate a model of adenovirus fiber binding to CAR [35] (Figure 7). The model was produced by superimposing JAM-A [24] and a full-length model of σ1 [14] onto the σ1H-D1 complex structure. Based on the positioning of σ1 and JAM-A in the model, JAM-A must reach beyond the approaching σ1 head to access residues in the C-terminal region of the σ1 tail. Residues in the predicted β-spiral repeat region of the σ1 tail, closer to the midpoint of the σ1 molecule, are required for engagement of carbohydrate [12]. Thus, the processes of JAM-A and carbohydrate engagement are likely facilitated by regions of flexibility within both the receptor and the viral attachment protein [8],[9],[24]. Since the binding sites for JAM-A are distinct from each other in the σ1 trimer, and since D1 projects from the cell surface, it is conceivable that each σ1 trimer simultaneously engages more than one JAM-A monomer. This scenario assumes that both monomers in the JAM-A dimer are located on the same cell. Binding of σ1 would lead to separation of JAM-A dimers into monomers, both of which likely remain in close proximity and could engage the same σ1 trimer. In this fashion, several molecules of JAM-A could form a clamp that engages σ1 and tightly adheres the virus to the cell, as depicted in our model.

Although the σ1 sequence is the most divergent among the reovirus proteins, prototype and field-isolate strains of the three most prevalent reovirus serotypes use JAM-A as a receptor [32]. Based on sequence alignment, the highest degree of conservation is observed among residues in the D–E loop, suggesting that this region forms part of the JAM-A-binding site [14],[32]. However, several T3D σ1 residues that interact with JAM-A are not conserved in prototype strains type 1 Lang (T1L) and type 2 Jones (T2J) σ1 [14]. For example, reovirus T2J possesses an alanine rather than an aspartate residue at position 423. We found that a mutant reovirus containing a D423A polymorphism exhibits reduced binding to JAM-A and diminished infectivity in HeLa cells in comparison to the parent virus (Figure 5A). These observations suggest that, while the binding sites may be similar, σ1-JAM-A interactions may differ at an atomic level among the reovirus serotypes. Serotype-specific differences such as the D423A polymorphism may in turn alter the affinity of σ1 proteins for JAM-A and thus influence reovirus tropism *in vivo*.

Structural analyses have revealed striking similarities between reovirus σ1 and adenovirus fiber and their respective receptors, JAM-A and CAR, pointing to an evolutionary relationship in the attachment strategies used by these viruses [36],[37]. A comparison of the σ1-JAM-A complex with that of the adenovirus type 12 (Ad12) fiber knob in complex with the D1 domain of human CAR [38] reveals conserved features, providing additional support for common ancestry among the two viruses. Both attachment proteins form trimers that bind three copies of the D1 domain of the receptor. Like JAM-A, CAR uses the dimer interface and the top (B–C and F–G loops) to engage its viral ligand. Also like JAM-A, fiber-contacting residues of CAR are mainly located in and adjacent to β-strands C, C′, C″, F, and G. Moreover, the thermodynamic properties of both interactions are remarkably similar. The K_D_ for the fiber-CAR complex is in the nanomolar range (0.5 to 1.5×10^−8^ M for Ad5 fiber [39]), which also is about 1,000-fold lower than the K_D_ of homodimeric CAR interactions (1.6×10^−5^ M [25]). However, unlike σ1, which uses sequences in the head and tail to bind JAM-A, the CAR-binding area in Ad12 fiber is located entirely in the knob and does not include residues in the shaft. In contrast to the σ1-JAM-A complex, in which one JAM-A D1 domain exclusively contacts one σ1 monomer, CAR also has some contacts with a second subunit in the fiber knob. Thus, the two virus-receptor complexes are similar in the contact areas formed by the receptors and the thermodynamic forces that contribute to complex formation, but the viral attachment proteins engage the receptors using different binding sites. Viruses in addition to adenovirus and reovirus engage CAR and JAM-A, respectively. Coxsackievirus binds CAR [40], and feline calicivirus binds fJAM-1, the feline homologue of JAM-A [5]. Both coxsackievirus and feline calicivirus, which are spherical nonenveloped viruses, require the D1 and D2 domains of their respective receptors for binding [41],[42]. The cryo-EM structure of feline calicivirus in complex with fJAM-1 shows that the virus binds both domains of fJAM-1 with more contacts located in the D1 domain [43]. Interestingly, the cryo-EM structure of coxsackievirus in complex with CAR shows that only the distal end of the D1 domain binds to the virus, but formation of complexes appears to require both CAR D1 and D2 [41].

The capacity to redirect viral vectors to specific target cells by modification of receptor-binding capacity provides a powerful approach for delivery of an engineered viral payload to an appropriate site. For example, retargeting adenovirus from cells expressing CAR to cells expressing JAM-A has been accomplished using a chimeric adenovirus that expresses reovirus σ1 in place of adenovirus fiber [44]. Development of plasmid-based reverse genetics for reovirus [7], coupled with the oncolytic potential of this virus [45]–[49], underscores the importance of a precise understanding of σ1 interactions with cellular receptors. Here, we provide proof-of-principle that reovirus mutants with structure-guided alterations in receptor-binding capacity can be engineered. This achievement represents a first step towards designing viruses containing modified σ1 proteins to target specific sites in the host based on receptor utilization.

The majority of known three-dimensional structures of viral proteins in complex with protein receptors involve molecules of the IgSF type. In addition to the complex presented here, such receptors are components of the HIV gp120-CD4 [50], rhinovirus-ICAM-1 [51], and adenovirus-CAR [38] complexes. In each case, the receptors exist as homodimers in solution [25],[52],[53] but are engaged as monomers by their viral ligands. For JAM-A and CAR, and possibly also for CD4 and ICAM-1, engagement by viruses is incompatible with the existence of a homodimer. Whether disruption of dimers alters cellular functions of these receptors is currently unclear. Although not an IgSF receptor, the recent crystal structure of ephrin-B2 bound to the Nipah virus G glycoprotein also shows that G engages an ephrin-B2 surface that normally interacts with the receptor Eph [54]. The σ1-JAM-A structure presented here may therefore reveal an ancient mechanism by which viruses usurp existing receptor interfaces and cleverly engage them in an energetically more favorable manner.

## Materials and Methods

### Protein Expression, Purification, and Complex Formation

Sequences corresponding to residues 28–129 of hJAM-A D1 (UniProtKB/Swiss-Prot entry Q9Y624) were amplified from a plasmid encoding full-length JAM-A [24] and cloned as an N-terminal GST-fusion into pGEX-4T-3 (GE Healthcare) using BamHI-XhoI restriction sites. The D1 E121A mutant was engineered from this construct [27]. JAM-A D1 and the T3D σ1 head domain (σ1H; residues 293–455; UniProtKB/Swiss-Prot entry P03528) were purified as described [13],[24], with minor modifications. Expression of the GST-D1 fusion proteins was induced in 1 L Luria Broth (Sigma-Aldrich) with 0.2 mM IPTG in *Escherichia coli* strain BL21 (DE3) pLysS (Novagen) at 25°C for 16 h. Bacteria were harvested by centrifugation, resuspended in 50 mM Tris [pH 7.5], 50 mM NaCl, 3 mM EDTA, 1% Triton X-100, 2 mM β-mercaptoethanol, 1 mM phenylmethylsulfonyl fluoride, and 100 µg/mL lysozyme, sonicated with 50% duty-cycle using a Branson Digital Sonifier 250, and centrifuged at 15,000×*g*. The clarified supernatant was passed over a 5 mL GSTrapFF column (GE Healthcare), which was washed with buffer (50 mM Tris [pH 7.5], 3 mM EDTA), ATP-Mg^2+^-buffer (20 mM MgSO_4_ and 10 mM ATP in buffer), and high-salt buffer (1 M NaCl in buffer). D1 was cleaved from GST on-column by overnight incubation with 150 units of thrombin (GE Healthcare) in 20 mM Tris [pH 7.8], 2.5 mM CaCl_2_, 150 mM NaCl. Induction of the σ1H construct was achieved using 0.4 mM IPTG, and bacteria were lysed using a high-pressure homogenizer (Avestin EmulsiFlex). After removal of GST, the sequence of each protein was identical to the native sequence with the exception of two amino acids at the N-terminus: Gly291 and Ser292 for σ1H and Gly26 and Ser27 for D1. None of these amino acids contribute to complex formation. Purified σ1H and D1 were mixed at a ratio of 1∶1.2 and incubated at 4°C for 30 min. Complexes were separated from excess D1 by size-exclusion chromatography in 20 mM Tris [pH 7.5], 100 mM NaCl using a Superdex 75 column (GE Healthcare). Analytical-scale size-exclusion chromatography to assay complex stability was performed using a SMART system (GE Healthcare) with a Superdex 75 PC 3.2/30 column.

### Crystallization and Structure Determination

The σ1H-D1 complex was concentrated to 4 mg/mL according to direct measurement of A_280_ and A_260_ (c[mg/mL] = 1.55×A_280_−0.76×A_260_). Crystals were initially obtained by mixing equal volumes of protein and 0.1 M CHES [pH 9.5], 30% polyethylene glycol 3000 (Wizard I Screen, Emerald BioSystems) at 20°C. Larger crystals were grown upon replacement of polyethylene glycol 3000 with polyethylene glycol 3350 and with streak seeding using cat whiskers (collected after natural loss). Crystals were flash-frozen with 20% glycerol as cryoprotectant. Data were collected at the X06SA beamline of the Swiss Light Source (Villigen, Switzerland) at 100 K and a wavelength of 0.92 Å using a MarCCD detector. The crystals were extremely thin. They had to be exposed for 10 seconds to an unattenuated beam to yield any diffraction beyond 4.0 Å and suffered severe radiation damage after only brief exposure. A total of 286 images from several dozen crystals were collected, and 85 of those were used to assemble the final data set. Since the radiation damage led to dramatic decreases in spot intensity for many reflections at higher resolution, we evaluated all processed data files with an in-house program, calculating the signal-to-noise ratio (*I/σI*) according to resolution bins for each frame in order to apply individual resolution cut-offs. This procedure significantly improved the overall quality of the data set.

Data were integrated and reduced with HKL (HKL Research). Crystals belong to the orthorhombic space group P2_1_2_1_2 (a = 105.9 Å, b = 124.3 Å, c = 130.6 Å). The asymmetric unit consists of two σ1H trimers, each complexed with three D1 monomers. Initial phases were obtained by molecular replacement with PHASER in CCP4 [55] using the trimeric T3D σ1H structure (PDB ID 2OJ5) [13] as a search model. Molecular replacement solutions for two σ1H trimers in the asymmetric unit were readily obtained and resulted in an overall *R*-factor of 40.1% (30–3.4 Å). Initial attempts to locate the D1 domains of hJAM-A (PDB ID 1NBQ) [24] by molecular replacement were not successful. However, 2F_obs_-F_calc_ and F_obs_-F_calc_ electron-density maps, calculated using phases obtained from the two σ1H trimers, which account for 61% of the protein atoms present in the crystal, clearly revealed the position and location of the six D1 domains. Adding the D1 domains to the structure reduced the overall *R*-factor to 34.7% (30–3.4 Å) before refinement.

The structure was refined using CNS [56] and Coot [57]. Refinement was performed using rigid body refinement, simulated annealing, restrained individual B-factor refinement, and conjugate gradient minimization. B-factors were refined individually because unrestrained group B-factor refinement was unstable. No sigma-cut-off was used. For the NCS restraints, we defined two groups of restrained coordinates. NCS group one contained all six copies of σ1, and NCS group two contained six copies of JAM-A D1. Thus, we did not restrain the complexes, but we did restrain the individual components, taking into account the partially dislodged D1 molecules (see results section). In all cases, loops that participate in crystal contacts and did not have the same structures in all copies were omitted from the restraining procedure. Electron-density maps were improved using non-crystallographic symmetry averaging [58] and data sharpening [59] by adding an overall B-factor of −70 Å^2^ to the observed structure factors with CAD [55]. Data sharpening improved some details in the electron density map and allowed us to resolve a number of side chains that had poor electron density prior to sharpening. However, the unsharpened map was traceable. Contact areas were calculated using AREAIMOL [55]. Coordinates and structure factors have been deposited with the Protein Data Bank with the accession code 3EOY. All structural figures were prepared using PyMOL [60].

### Size-Exclusion Chromatographic Analysis of Complex Stability

The effect of pH on complex stability was investigated by concentrating purified σ1H, wt D1, monomeric D1 E121A [27], and the σ1H-D1 complex to 10% of the original volume using Millipore 5,000 MWCO filters. Samples were diluted in 20 mM citrate buffers [pH 4.0, 4.5, or 5.0] or 20 mM Hepes [pH 7.4] and re-concentrated. This procedure was repeated five times. Size-exclusion chromatography was performed using the respective buffer for each sample, containing 100 mM NaCl.

### Analytical Ultracentrifugation

For analytical ultracentrifugation experiments, JAM-A D1 was subjected to size-exclusion chromatography using a Superdex 75 column in 20 mM Tris [pH 7.5], 100 mM NaCl. Sedimentation velocity and equilibrium experiments were performed at 25°C using a BeckmanCoulter (Krefeld, Germany) Xl-I analytical ultracentrifuge equipped with interference optics. The solvent density and partial specific volume of JAM-A D1 were calculated from composition using known density increments. Two-sector titanium centerpieces of 12 mm or 20 mm optical pathlengths (Nanolytics, Germany) were employed. A factor of 3.29 mg/mL/fringes was used to convert signal units into molar quantities. For sedimentation velocity experiments, 400 µL of protein solution at five concentrations between 0.06 and 1.31 mg/mL were centrifuged at 50 krpm. The concentration profiles were scanned every two minutes until all material had sedimented. Data were evaluated using the c(s)-function implemented in SedFit, version 9.4 [61]. For sedimentation equilibrium experiments, four initial concentrations between 1.6–0.16 mg/mL were prepared, and 150 µL of these solutions were centrifuged at three different velocities (17.5/25/35 krpm). Attainment of apparent sedimentation and chemical equilibrium was verified using MATCH. Equilibrium gradients were globally analyzed using NonLin (MATCH and NonLin are available at http://www.biotech.uconn.edu/auf/?i=aufftp). Suitable models to describe the experimental data were selected based on minimized variance and visual inspection of the residuals run pattern. Different initial starting values for the floated parameters were used to confirm that the parameters were well defined by the data.

### Cells, Viruses, and Antibodies

HeLa cells were propagated as described [31]. Reovirus strain rsT3D-σ1T249I (parent) was engineered using plasmid-based reverse genetics [7]. Reoviruses were purified by cesium chloride-gradient centrifugation from infected L cells [9]. Particle concentrations were determined using the conversion factor 1 AU_260_ = 2.1×10^12^ particles. Titers of virus stocks were determined by plaque assay using L cells [62]. Attenuated vaccinia virus strain rDIs-T7pol expressing T7 RNA polymerase was propagated using chick embryo fibroblasts [63].

### Plasmid-Based Reovirus Rescue

The parental *S1* gene used for these studies encodes a σ1 molecule with a threonine to isoleucine substitution at position 249, which renders σ1 resistant to proteolytic cleavage [7]. Substitution mutations were engineered in pBacT7-S1T3D T249I [7] using QuickChange site-directed mutagenesis (Stratagene). Reoviruses were recovered from plasmids as described [7]. Mutations in the *S1* gene were confirmed using the OneStep RT-PCR kit (Qiagen), gene-specific primer sets, and viral dsRNA extracted from infected L cells as template. Purified PCR products were directly subjected to sequence analysis.

### Reovirus Infectivity in HeLa Cells

HeLa cells (2×10^5^/well) were plated in 12-well plates and incubated at 37°C overnight. Cells were treated with either phosphate-buffered saline (PBS) alone (mock) or 40 mU/ml of *Arthrobacter ureafasciens* neuraminidase (MP Biomedicals, LLC) diluted in PBS at 37°C for 1 h prior to adsorption with reovirus at an MOI of 50 PFU/cell. Following incubation at 25°C for 1 h, cells were washed with PBS and incubated at 37°C for 18–20 h. Infected cells were processed for indirect immunofluorescence as described [31]. Images were captured at 200× magnification using a Zeiss Axiovert 200 microscope. For each experiment, three fields of view were scored. Mean values from three independent experiments were compared using the unpaired student's *t* test as applied using Microsoft Excel. *P* values of less than 0.05 were considered statistically significant.

### Reovirus Binding Assays

A BIAcore CM5 chip (GE Healthcare) was coated with mouse ascites containing monoclonal GST-specific antibody (Sigma) to ∼1800 resonance units by amine coupling. Purified GST or GST-JAM-A ectodomain fusion proteins at a concentration of 2 µM in HEPES-buffered saline [pH 7.0] were captured by injection across individual flow cells of an antibody-coated chip for 2.5 minutes at 20 µL/min using a BIAcore 2000 (GE Healthcare). Purified parent or mutant reovirus (6×10^12^, 8×10^12^, and 10^13^ particles/mL) was injected across the conjugated chip surface at 20 µL/min. Following reovirus binding, chip surfaces were regenerated with a 20 µL pulse of 10 mM glycine [pH 2.5]. Data analysis was performed using BIAevaluation 3.0 software (GE Healthcare).

## Supporting Information

Figure S1 Real space correlation plots. Real space correlation plots [66] (black) and *B*-factor plots (blue) for a single σ1H chain (top) and a D1 chain (bottom). Some regions participating in contacts are shaded. The asterisk indicates the position of the 3_10_ helix. Plots were calculated at the TB consortium bias removal server (http://tuna.tamu.edu).
(0.12 MB TIF)

Figure S2 Ultracentrifugation experiments. (A) Sedimentation velocity experiments. Sedimentation coefficient (c(s)) distributions, with c(s) as the concentration of species with sedimentation coefficients between *s* and *s*+*ds* for five concentrations of JAM-A D1. Little change in the sedimentation coefficient of the main component around 2.35 S is observed. The small additional peak seen in variable amounts around 3.8 S likely corresponds to JAM-A D1 tetramers. The curves have been normalized to a total area of unity and offset for clarity. Note that the exact shape of the c(s)-traces depends on the signal-to-noise ratio and the detailed structure of the systematic noise from the interference data. (B) Sedimentation equilibrium results for JAM-A D1. Top panel: Raw experimental data for 17.5/25/35 krpm (black, red, and green dots, respectively) at 0.8 mg/mL together with the theoretical curves for a monomer-dimer-equilibrium (solid black lines) from which the equilibrium coefficient was derived (see text). For clarity, only every 5th data point is displayed for only one starting concentration (of four). Bottom panel: Local deviations between theoretical and experimental curves. All data points are shown. Residuals were offset by a constant factor of 0.1 for clarity.
(0.48 MB TIF)
