# Supplementary figures and images for: Structure of Reovirus σ1 in Complex with Its Receptor Junctional Adhesion Molecule-A
Source: PLoS Pathog. 2008 Dec 12;4(12):e1000235. doi: 10.1371/journal.ppat.1000235 (PMC2588538; doi:10.1371/journal.ppat.1000235)

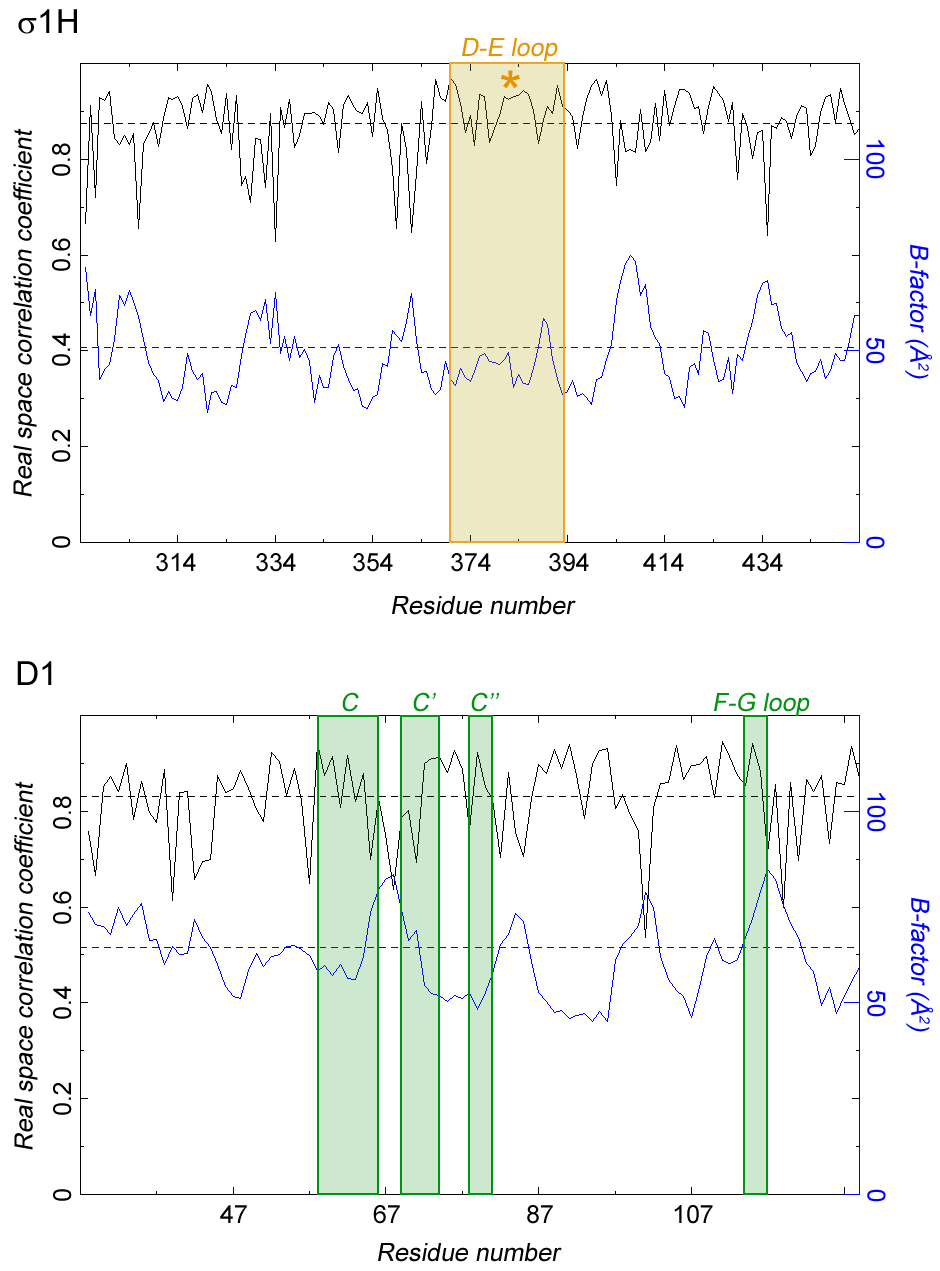

Supplement: Figure S1 — Real space correlation plots. Real space correlation plots [66] (black) and B-factor plots (blue) for a single σ1H chain (top) and a D1 chain (bottom). Some regions participating in contacts are shaded. The asterisk indicates the position of the 310 helix. Plots were calculated at the TB consortium bias removal server (http://tuna.tamu.edu). (0.12 MB TIF) [file ppat.1000235.s001.tif]

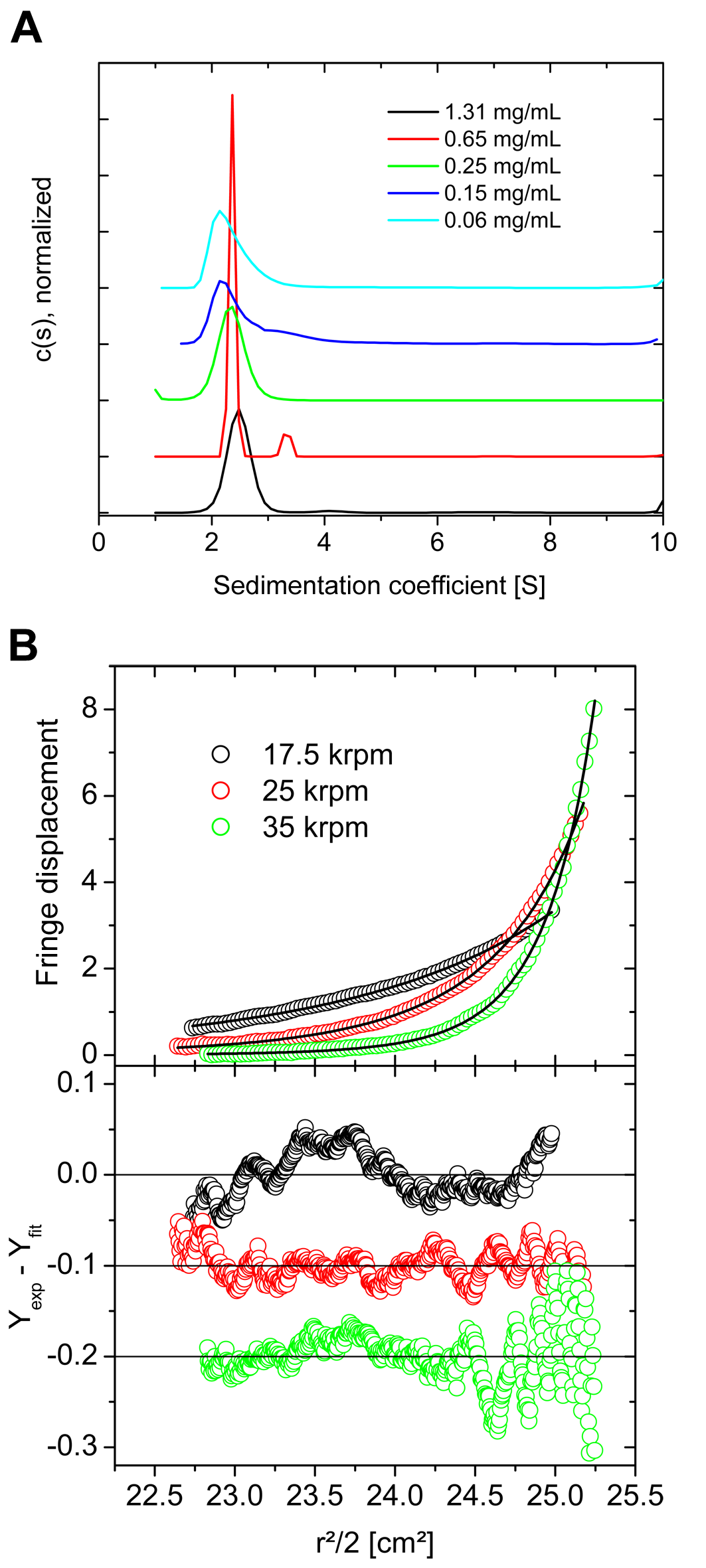

Supplement: Figure S2 — Ultracentrifugation experiments. (A) Sedimentation velocity experiments. Sedimentation coefficient (c(s)) distributions, with c(s) as the concentration of species with sedimentation coefficients between s and s+ds for five concentrations of JAM-A D1. Little change in the sedimentation coefficient of the main component around 2.35 S is observed. The small additional peak seen in variable amounts around 3.8 S likely corresponds to JAM-A D1 tetramers. The curves have been normalized to a total area of unity and offset for clarity. Note that the exact shape of the c(s)-traces depends on the signal-to-noise ratio and the detailed structure of the systematic noise from the interference data. (B) Sedimentation equilibrium results for JAM-A D1. Top panel: Raw experimental data for 17.5/25/35 krpm (black, red, and green dots, respectively) at 0.8 mg/mL together with the theoretical curves for a monomer-dimer-equilibrium (solid black lines) from which the equilibrium coefficient was derived (see text). For clarity, only every 5th data point is displayed for only one starting concentration (of four). Bottom panel: Local deviations between theoretical and experimental curves. All data points are shown. Residuals were offset by a constant factor of 0.1 for clarity. (0.48 MB TIF) [file ppat.1000235.s002.tif]
